# Supplementary material for: Comparative Evaluation of Chemical Composition and Nutritional Characteristics in Various Quinoa Sprout Varieties: The Superiority of 24-Hour Germination
Source: Foods. 2024 Aug 12;13(16):2513. doi: 10.3390/foods13162513 (PMC11353781; doi:10.3390/foods13162513)
Supplement: Supplementary file 1 [file foods-13-02513-s001.zip › foods-3125847-supplementary.pdf]

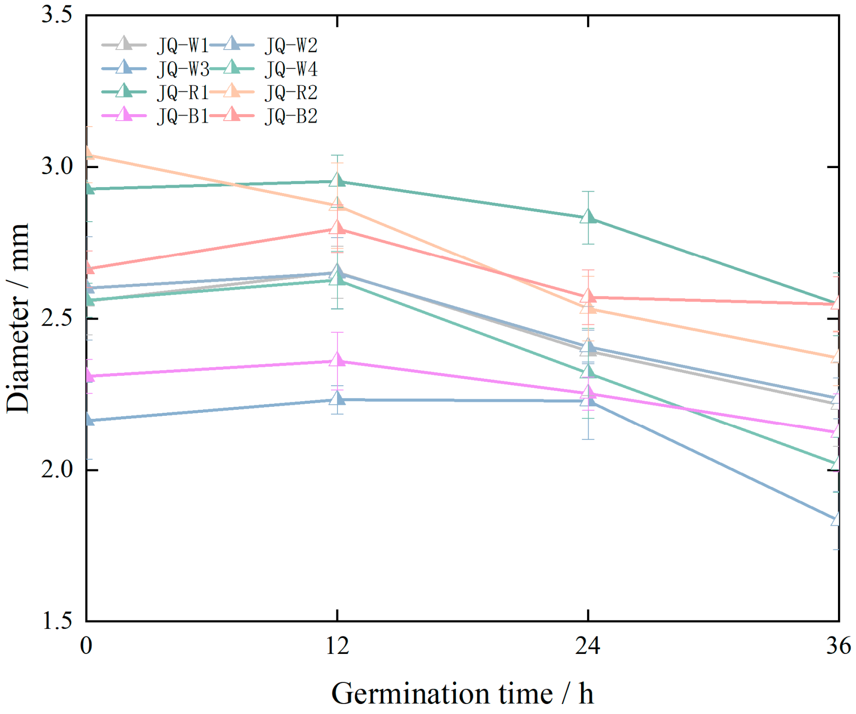

Figure S1. The diameter of quinoa from seeds to sprouts at different germination time points. The data at each time point in the figure are mean ± SD, n = 3.

Table S1. Relationship analysis of seed weight, hypocotyl length, and seed diameter with germination time and varieties.

Time	Sample	Thousand weight (g)	Length of the hypocotyl (mm)	Diameter (mm)
0h	JQ-W1	4.20±0.37cd***	nd.	2.56±0.11b
	JQ-W2	4.87±0.53bc**	nd.	2.60±0.17b
	JQ-W3	3.55±0.09d***	nd.	2.16±0.13c
	JQ-W4	5.04±0.53b*	nd.	2.56±0.06b
	JQ-R1	6.88±0.26a***	nd.	2.93±0.11a
	JQ-R2	6.50±0.13a***	nd.	3.04±0.09a
	JQ-B1	3.69±0.13d***	nd.	2.31±0.06c
	JQ-B2	4.77±0.74bc**	nd.	2.66±0.06b
12h	JQ-W1	6.51±0.15d	5.71±0.16c	2.65±0.09b
	JQ-W2	7.86±0.61c	9.61±0.13a	2.65±0.12b
	JQ-W3	4.83±0.04e	5.20±0.17d	2.23±0.05c
	JQ-W4	6.14±0.26d	5.90±0.14c	2.63±0.10b
	JQ-R1	9.78±0.4a	9.60±0.16a	2.95±0.09a
	JQ-R2	8.64±0.19b	7.56±0.31b	2.87±0.14a
	JQ-B1	5.10±0.10e	4.40±0.14e	2.36±0.10c
	JQ-B2	7.47±0.4c	7.48±0.18b	2.80±0.08ab

24h	JQ-W1	8.38±0.26bc***	14.57±0.73c***	2.39±0.15bc
	JQ-W2	8.13±0.38c	18.48±0.67a***	2.41±0.06bc*
	JQ-W3	6.65±0.44d**	10.73±0.43d***	2.23±0.24c
	JQ-W4	8.07±0.39c**	17.11±0.38b***	2.32±0.15bc*
	JQ-R1	11.04±0.29a**	10.64±0.25d**	2.83±0.09a
	JQ-R2	10.29±1.04a	14.88±0.42c***	2.53±0.11b*
	JQ-B1	6.03±0.27d**	11.55±0.70d***	2.25±0.06c
	JQ-B2	9.15±0.44b**	9.23±0.32e**	2.57±0.09b*
36h	JQ-W1	8.91±0.33d***	19.62±0.63c***	2.22±0.14bcd*
	JQ-W2	9.23±0.16cd*	22.98±0.22b***	2.24±0.07bc**
	JQ-W3	7.01±0.13e***	18.22±0.39***	1.83±0.10e**
	JQ-W4	9.88±0.52c***	18.53±0.58cd***	2.02±0.09de**
	JQ-R1	11.95±0.6a**	16.51±1.16e***	2.55±0.10a*
	JQ-R2	10.97±0.44b**	27.43±0.91a***	2.37±0.09ab**
	JQ-B1	7.51±0.28e***	15.06±0.7f***	2.12±0.13cd
	JQ-B2	11.63±0.85ab**	19.34±0.47cd***	2.55±0.09a*

Lowercase letters signify significance among different varieties simultaneously, with $p < 0.05$.The * indicates significance within the same variety at different times, using the 12-hour value as the reference point, where * denotes $p < 0.05$, ** denotes $p < 0.01$, and *** denotes $p < 0.001$.The data at each time point in the table are mean ± SD, n = 3.

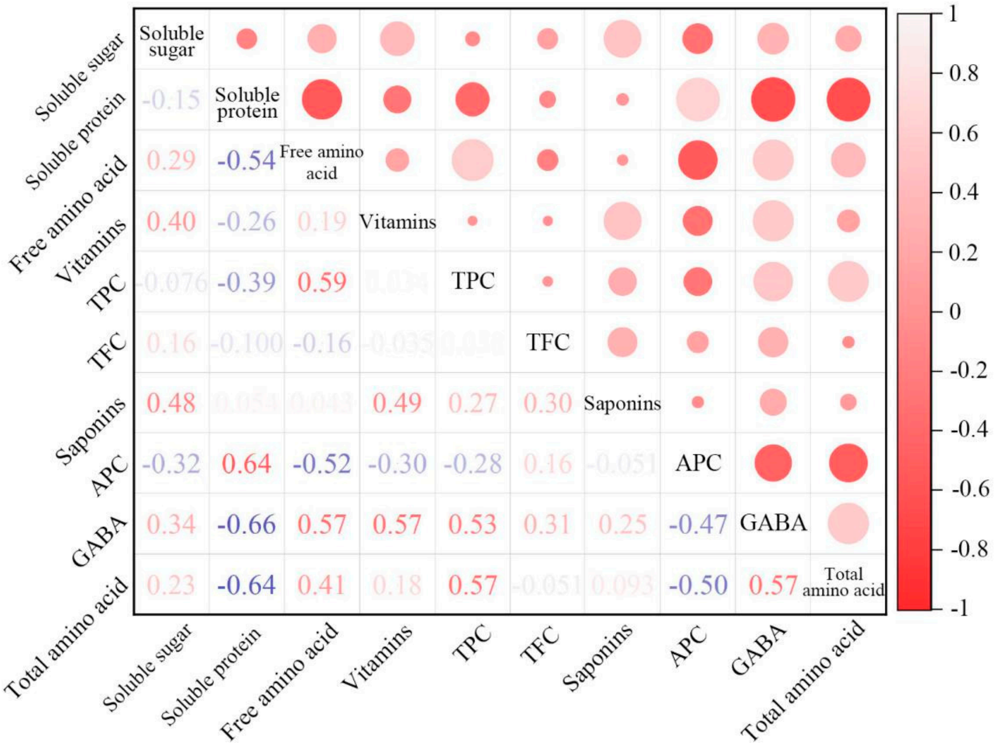

Figure S2. Correlation analysis of the content of APC, TFC, etc., in quinoa seedlings of different varieties at various time points. n = 3, p<0.05.

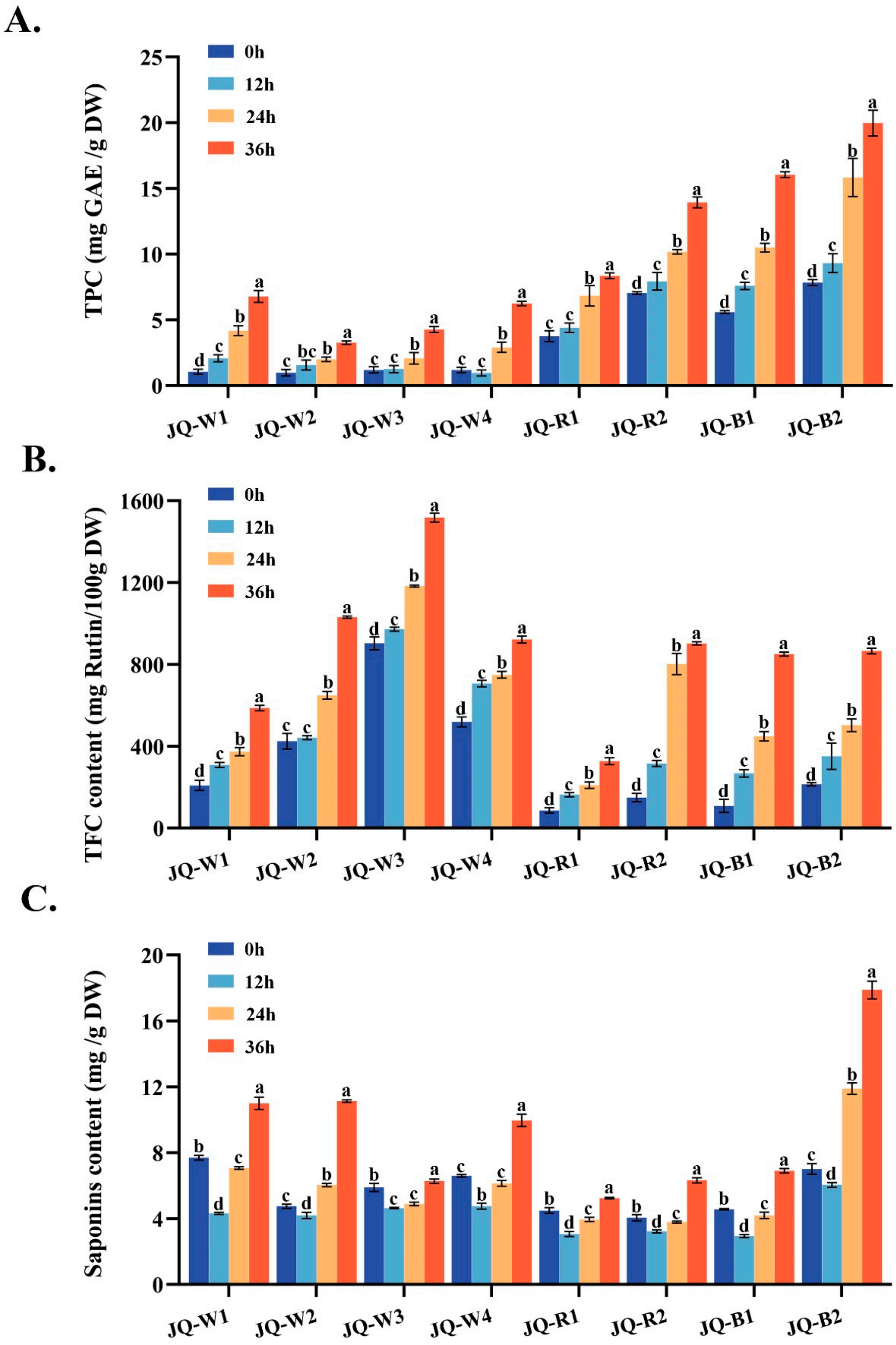

Figure S3. TPC (Total phenols content) (A), TFC (total flavonoids content) (B), and Saponins (C) content of quinoa sprouts at different time points. The data at each time point in the table are mean ± SD, n = 3.
